# Glycosylated RNAs: discovery and implications in cancer biology

**DOI:** 10.3389/fonc.2026.1719799

**Published:** 2026-02-04

**Authors:** Shuai Tang, Ximo Wang, Fangmin Chen, Benyi Li

**Affiliations:** 1 Department of Urology, Tianjin Third Central Hospital, Tianjin, China; 2 Department of Urology, The University of Kansas Medical Center, Kansas, KS, United States; 3 College of Medicine, Nankai University, Tianjin, China; 4 Department of Urology, Wuhan Sixth Hospital, Wuhan, China

**Keywords:** cancer, cell surface biomarker, extracellular vesicles, glycoRNAs, tumor microenvironment

## Abstract

Glycosylated RNAs (glycoRNAs) are a newly discovered class of biomolecules that challenge the long-standing paradigm that glycosylation occurs exclusively on proteins and lipids. Early studies indicate that glycoRNAs are broadly distributed across cell types and can be detected at the cell surface as well as in other cellular compartments. Emerging evidence suggests that glycoRNAs may participate in processes such as intercellular communication and immune regulation, but their context-dependent functions in physiology and disease, including cancer, are only beginning to be elucidated. Recent advances in detection techniques have enabled more comprehensive profiling of glycoRNAs and their associated cell-surface RNA-binding proteins, providing initial links between glycoRNA patterns, tumor aggressiveness, immune checkpoint regulation and extracellular vesicle–mediated signaling. However, many of these connections remain correlative or are inferred by analogy to protein and lipid glycosylation. In this review, we summarize current knowledge of glycoRNA biosynthesis, cell-surface display and detection methods, with a particular focus on emerging observations in cancer-related contexts. We also discuss the potential of glycoRNAs as biomarkers and therapeutic targets, while highlighting key unanswered questions regarding their biosynthetic pathways, structural diversity and mechanistic roles. Addressing these challenges with integrated omics and spatial approaches will be essential for defining glycoRNA biology and evaluating their feasibility as tools for precision oncology.

## Introduction

Glycosylated RNAs (glycoRNAs), a newly discovered class of biomolecules in 2021 by the Bertozzi lab, represent a significant advance in the fields of RNA biology and glycobiology (1). This discovery overturns the conventional paradigm that glycosylation occurs exclusively in proteins and lipids. GlycoRNAs are RNAs covalently modified with glycans, with a fraction detectable on the cell surface as well as in other compartments. Because many mechanistic insights into glycoRNAs currently come from non-malignant or inflammatory models, we include these systems when they illuminate processes—such as leukocyte recruitment, epithelial barrier regulation and extracellular vesicle communication—that are also fundamental to tumor biology. These examples are therefore discussed for their conceptual relevance to cancer rather than as direct evidence from tumor models. In particular, their activities within the tumor microenvironment have attracted growing interest, as glycoRNAs have been proposed to influence tumor immune interactions, metastatic behavior, and therapy responses (2). However, direct causal evidence for these roles is still sparse, and further mechanistic studies will be required to establish how glycoRNAs contribute to cancer biology.

## 1 Discovery and biosynthesis of glycoRNAs

GlycoRNAs are widely distributed across various cell types and tissues, where they participate in regulating the transmission of diverse and essential biological information. The first report of glycoRNAs was published in 2021, showing that small non-coding RNAs, including Y-RNAs, snRNAs, rRNAs, snoRNAs, and tRNAs, can undergo N-linked glycosylation. Notably, although numerous types of chemical modifications have been identified in messenger RNAs within the epitranscriptome, no glycosylated mRNAs have been discovered to date. Instead, glycosylation predominantly occurs in small RNA species, with Y-RNAs representing the largest proportion (1).

The glycan structures identified in glycoRNAs are highly like the sialylated glycans commonly observed in protein glycosylation. The biosynthetic mechanisms of glycoRNAs remain incompletely understood but are hypothesized to involve canonical glycosyltransferases (such as GALNTs and sialyltransferases) as well as oligosaccharyltransferase (2). Current studies suggest that N-linked glycosylation of RNAs may involve components of the oligosaccharyltransferase (OST) complex, including STT3A, whereas the role of the DTWD2 gene and the exact trafficking route remain to be fully established (3, 4). Recently, two classes of glycoRNAs were identified in human monocytes (THP-1 cells and peripheral blood mononuclear cells): glycoRNA-L (~11 kb) and glycoRNA-S (~0.6 kb). Both species are susceptible to RNase digestion, confirming their cell surface localization. Functional analyses revealed that glycoRNAs regulate monocyte-endothelial adhesion by interacting with Siglec-5, a critical step in inflammatory cell infiltration (5). Although these data were obtained in non-malignant inflammatory and epithelial models, monocyte adhesion and transmigration are also key features of tumor-associated vasculature and leukocyte recruitment. For this reason, we summarize these findings here for their relevance to microenvironmental processes that are likewise important in cancer, even though direct glycoRNA mechanisms have not yet been demonstrated in tumor models.

Contrary to the traditional notion that RNAs are confined to the nucleus and cytoplasm, glycoRNAs can also localize to the cell surface and modulate gene expression through interactions with RNA-binding proteins (RBPs). Potential sources of cell surface RNAs (csRNAs) include: (i) secretion *via* extracellular vesicles; (ii) translocation of ribonucleoprotein complexes; (iii) membrane anchoring through glycan or lipid modifications; and (iv) N-glycosylation mediated by the endoplasmic reticulum-Golgi pathway. These mechanisms may act synergistically to generate the diverse origins of csRNAs (6, 7). Importantly, not all csRNAs are glycosylated, and not all glycoRNAs reside at the cell surface; some glycoRNAs are detected in intracellular compartments or within vesicles.

Furthermore, studies have shown that PNGaseF treatment or depletion of acp³U biosynthetic enzymes only modestly reduced rPAL signals, suggesting that most sialylated RNA signals are not derived from N-glycans. This observation has led to the hypothesis that O-glycans also contribute to RNA glycosylation. Selective enrichment and analysis workflows have demonstrated O-glycoRNA capture and profiling in cellular samples (8, 9), additional preliminary evidence has been reported (preprint) (10). In addition, many RBPs have been detected on the cell surface (referred to as cell-surface RBPs, or csRBPs) and form clustered structures with glycoRNAs. This discovery suggests that the cell surface may serve as a novel platform for RNA-RBP interactions, with potential implications in cancer biology, immune regulation, and autoimmune diseases (11).

## 2 Methods for detecting glycoRNAs

The discovery of glycoRNAs was achieved using a metabolic labeling strategy. In this approach, monosaccharide precursors carrying azide groups (e.g., Ac_4_ManNAz, FucAz) were incorporated into newly synthesized glycans, which were then coupled to fluorescent or biotin probes *via* click chemistry, enabling specific labeling and detection of glycoRNAs (1). Subsequent enzymatic treatments provided further validation: PNGaseF digestion abolished the signal, whereas O-glycosidase treatment had no effect, confirming the presence of N-linked glycosylation. Lectin-binding assays were then employed to characterize specific glycan structures, including sialic acid and fucose residues (12). However, metabolic labeling requires active precursor uptake and *de novo* glycan synthesis and cannot be readily applied to fixed tissues or archival clinical samples. In addition, azido-sugar incorporation labels all newly synthesized sialoglycans and fucosylated glycans, including those on glycoproteins and glycolipids; therefore, careful controls are needed to ensure that detected signals indeed arise from covalently glycosylated RNAs rather than from RNAs non-covalently associated with labeled glycoproteins.

The RNA-optimized Periodate Oxidation and Aldehyde Labeling (rPAL) method was recently developed to enable the sensitive detection of glycosylated RNAs. The principle of rPAL relies on the selective oxidation of vicinal diols present on sialic acids by sodium periodate (NaIO_4_), which generates reactive aldehyde groups. Compared with the ribose 2′,3′-diols of RNA, the 7,8-diol moieties of sialic acids exhibit much higher reactivity under mild conditions (13), thereby allowing preferential labeling of glycans rather than the RNA backbone. The resulting aldehydes can then react with aminooxy probes such as aminooxy-biotin to form stable oxime bonds, enabling covalent labeling and subsequent enrichment of glycoRNAs (14). This strategy provides more than 25-fold higher sensitivity compared with traditional metabolic labeling approaches and eliminates the need for metabolic precursor incorporation (15), making it particularly suitable for analyzing endogenous glycoRNAs in native tissues and clinical samples. At the same time, rPAL relies on chemical oxidation and affinity capture, which can potentially oxidize other vicinal diols and co-enrich RNAs that are tightly associated with glycoproteins. For this reason, extensive enzymatic controls and orthogonal validation are required to distinguish truly covalently glycosylated RNAs from RNAs that merely co-purify with oxidized glycoconjugates.

A sialic acid aptamer and RNA *in situ* hybridization-mediated proximity ligation assay (ARPLA) was subsequently established to visualize glycoRNAs on the cell surface (16). This method relies on a dual-recognition strategy: a Neu5Ac aptamer selectively binds to the glycan moiety of glycoRNAs, while an RNA *in situ* hybridization (RISH) probe targets their RNA sequence. When both probes simultaneously bind to the same glycoRNA molecule, their linker regions are brought into proximity, allowing T4 DNA ligase to circularize a connector oligonucleotide. The circular DNA then serves as a template for rolling circle amplification (RCA) catalyzed by phi29 polymerase, generating long single-stranded DNA products that can hybridize with fluorescent probes to produce amplified imaging signals. This dual-recognition mechanism ensures high specificity, reducing false positives that may arise from detecting glycans or RNA alone (17). Another study proposed a galactose oxidase-glycosidase strategy to analyze glycoRNAs (9). By oxidizing glycan moieties and applying specific glycosidases, the method enables validation of N-linked glycoRNAs under mild conditions that preserve RNA integrity. Combined with RNA-seq and LC-MS/MS, it directly profiles endogenous glycoRNAs, particularly small noncoding RNAs, offering higher specificity than metabolic labeling, though its utility is limited by RNA stability during sequential oxidation and digestion steps and by the need for stringent controls to avoid off-target oxidation.

Beyond N-glycosylation, a solid-phase chemoenzymatic method, TnORNA, was designed for the specific capture and enrichment of O-glycosylated RNAs (O-glycoRNAs) (8). The TnORNA (solid-phase chemoenzymatic Tn-glycoRNA method) was developed to specifically detect and enrich O-glycosylated RNAs, a modification that had been difficult to study due to the lack of selective tools. Its principle is based on the unique structural feature of O-glycans-particularly the Tn antigen (N-acetylgalactosamine, GalNAc). By exploiting this characteristic, the method uses a combination of solid-phase chemistry and enzymatic reactions to capture O-glycoRNAs from complex RNA mixtures. This approach distinguishes itself from earlier techniques (such as rPAL and ARPLA, which primarily detect N-linked glycoRNAs) by providing direct and selective identification of O-linked modifications. However, TnORNA is inherently biased toward Tn-type O-glycans and may miss glycoRNAs carrying other O-linked structures. In addition, solid-phase handling steps can introduce losses or favor more abundant species, so quantitative interpretation needs to be made with caution.

Recent advances have enabled direct detection of glycosylated RNAs (glycoRNAs) on the surface of alveolar epithelial cells using a combination of chemical and imaging-based approaches (18). By metabolically incorporating azide-modified monosaccharides such as Ac4ManNAz and FucAz, newly synthesized sialylated and fucosylated glycoRNAs could be labeled through copper-free click chemistry with DBCO-Sulfo-Cy5, providing a dynamic view of *de novo* glycoRNA expression. Compared with earlier bulk RNA-based methods such as rPAL or ARPLA, this integrated strategy offers higher specificity for cell-surface RNAs, improved sensitivity through in-gel imaging without transfer losses, and the ability to distinguish newly formed from pre-existing glycoRNAs. While these observations were obtained in non-cancer epithelial contexts, similar epithelial and stromal compartments are key components of many solid tumors. We therefore discuss these studies here because glycan-dependent exRNA and glycoRNA packaging in such cells is likely to inform future work on tumor–host communication, even though direct evidence in cancer models is still lacking. A concise comparative overview of these and related detection or profiling approaches is presented in **Table 1**.

**Table 1:** Summary of glycoRNAs detection methods.

Method	Principle	Advantages	Limitations	Applications	Reference
Metabolic labeling + Click chemistry	Azido-sugars (Ac_4_ManNAz, FucAz) incorporated into glycans, coupled to DBCO probes via click chemistry.	Enables visualization of nascent glycoRNAs; compatible with imaging.	Requires metabolic incorporation; unsuitable for fixed tissues.	Dynamic labeling and detection of sialylated/fucosylated glycoRNAs.	(1)
rPAL	Oxidation of sialic acid diols → aldehyde labeling with aminooxy-biotin.	No metabolic labeling needed; high sensitivity.	Requires careful oxidation control; biased to sialoglycoRNAs.	Enrichment and LC-MS/MS identification of endogenous glycoRNAs.	(15)
ARPLA	Neu5Ac aptamer + RNA probe trigger ligation and RCA amplification.	High specificity; single-cell imaging.	Probe design and workflow are complex.	Visualization and localization of surface glycoRNAs.	(16)
Galactose oxidase–glycosidase	Oxidation and enzymatic digestion to validate N-linked glycoRNAs.	Preserves RNA integrity; compatible with omics.	Glycan-type dependent; multi-step.	Verification of N-linked glycoRNAs.	(9)
TnORNA	Solid-phase capture and enzymatic release based on Tn antigen (GalNAc).	Specific for O-glycoRNAs; robust enrichment.	Specialized workflow; limited sample types.	O-glycoRNA enrichment and identification.	(8)

Metabolic labeling can be coupled to fluorescence imaging for *in situ* visualization of cell-surface glycoRNAs in live or fixed cells (18).

To date, most studies identify or visualize glycoRNAs using biochemical enrichment and imaging rather than sequencing. Because conventional RNA-seq relies on cDNA libraries, glycan features—monosaccharide composition, linkage type, and branching—are not represented in the sequence readout, resulting in limited base- or site-level assignment of glycoRNAs (19, 20). By contrast, nanopore direct RNA sequencing interrogates native RNA molecules and, with ongoing advances in chemistry and modeling, can detect several canonical RNA modifications (21). However, peer-reviewed applications specifically targeting glycoRNAs have not yet been reported. Accordingly, the interpretation of glycoRNA datasets still requires careful consideration of each method’s limitations, and further methodological refinement will be essential to unambiguously define the chemical nature and sites of RNA glycosylation.

## 3 GlycoRNAs and lipid rafts

Recent single-cell imaging using ARPLA—a dual-recognition proximity ligation assay that detects sialylated glycans and their cognate RNA sequences—has revealed that a substantial fraction of cell-surface glycoRNA signals colocalize with cholesterol- and sphingolipid-rich membrane microdomains (“lipid rafts”). In addition to mapping surface distributions, ARPLA-based studies reported glycoRNA trafficking along secretory pathways, suggesting that microdomain organization may facilitate presentation and turnover at the plasma membrane (16). These observations position lipid rafts as a spatial scaffold that could increase encounter rates between glycoRNAs, and raft-resident receptors or adhesion molecules implicated in immune regulation.

Notably, cationic cell-penetrating motifs such as the HIV-1 Tat basic domain—widely used as raft-targeting or raft-sensitive probes—traffic through heparan-sulfate–dependent endocytosis and accumulate in lipid raft microdomains (22). Prior work shows that the Tat basic domain is essential for raft targeting and signaling outputs, and that arginine-rich CPPs can induce proteoglycan clustering and recruitment to actin-associated lipid rafts (23, 24). Functionally, raft organization is known to tune receptor signaling and adhesion in cancer and immune cells; placing glycoRNAs in this context offers a parsimonious explanation for their reported roles in neutrophil recruitment and immune modulation (25). Because glycoRNAs and their associated cell-surface RNA-binding proteins (csRBPs) reside on the outer leaflet of the plasma membrane, it is reasonable to ask whether they also partition into these cholesterol-rich regions. At present, however, direct quantitative evidence for glycoRNA partitioning into biochemically defined lipid rafts and for raft-dependent functional effects remains limited. Many of the mechanistic ideas about how rafts might regulate glycoRNAs—for example, by concentrating Siglec ligands, coordinating co-receptors or coupling glycoRNA display to extracellular vesicle budding—are inferred by analogy to glycoprotein and glycolipid signaling rather than demonstrated experimentally.

## 4 Cell surface RNA-binding proteins as biomarkers

GlycoRNAs exhibit tumor specificity and hold promise as biomarkers for early diagnosis, prognosis evaluation, and therapeutic response monitoring. Alterations in glycosylation patterns often occur during the early stages of tumorigenesis. Distinct cancer-associated glycosylation signatures have been identified in precancerous lesions across multiple cancer types, making them effective diagnostic indicators. Clinically approved glycoproteins such as AFP (for hepatocellular carcinoma), CEA (for colorectal cancer), and PSA (for prostate cancer) exemplify the utility of glycosylation-based biomarkers (26–28). The classical secretory pathway (*via* the endoplasmic reticulum and Golgi apparatus) represents the major source of cell-surface proteins, which are typically glycosylated. Since RNA is generally not localized within the ER or Golgi, it has been hypothesized that RNA glycosylation may rely on the chaperone functions of RNA-binding proteins (RBPs) or occur through non-canonical transport routes that allow transient contact with glycosylation enzymes (2). In 2025, a comprehensive review described the identification, organization, and functions of cell-surface RNA-binding proteins (csRBPs) in cell communication and endocytosis. Cell-penetrating peptides (CPPs), such as the HIV-TAT peptide, have traditionally been thought to enter cells primarily by interacting with heparan sulfate proteoglycans (HSPGs) due to their positively charged properties. However, recent studies revealed a novel mechanism: TAT is internalized through binding to clusters of glycoRNA-csRBP complexes on the cell surface (29).

**Figure 1:**
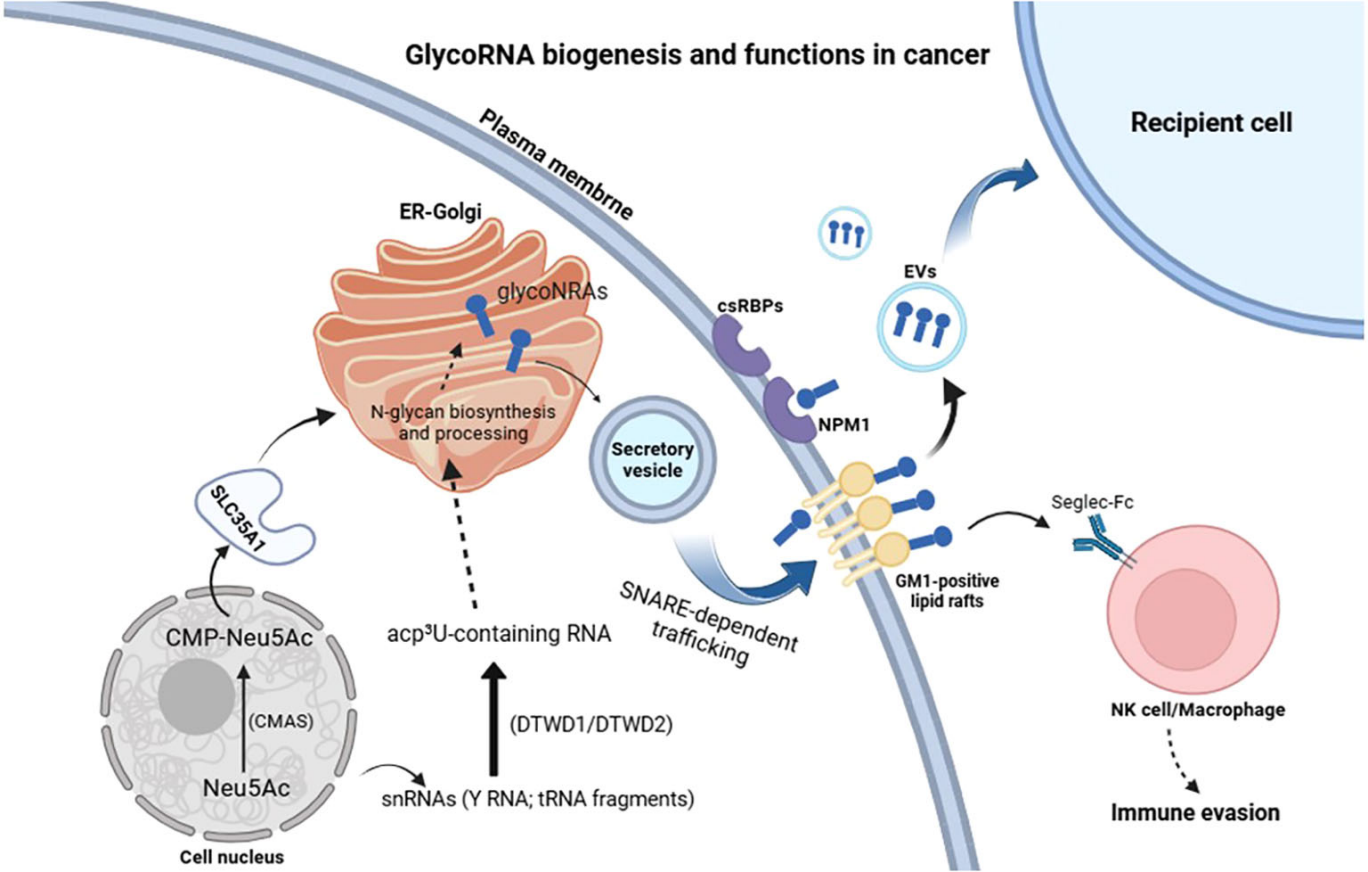
Proposed model of glycoRNA biogenesis, trafficking and functions in cancer. Small noncoding RNAs such as Y RNAs, tRNA fragments and snoRNAs represent major RNA species that can carry glycan modifications and thus constitute the currently known glycoRNAs. Following transcription, glycoRNAs undergo glycosylation within the ER–Golgi network through the CMAS–SLC35A1 enzymatic pathway. GlycoRNAs are then transported via intracellular vesicles to the plasma membrane, where they interact with Siglec receptors and form clusters with cell-surface RNA-binding proteins (csRBPs), for example NPM1. These surface-localized glycoRNAs participate in immune regulation, cell signaling, and adhesion. A subset of glycoRNAs and csRBPs resides in cholesterol-rich lipid rafts, promoting nanocluster formation, Siglec engagement, and coupling to EV trafficking. Solid arrows denote steps supported by experimental data, whereas dashed arrows highlight proposed or unresolved aspects of glycoRNAs biogenesis and function, particularly in the context of cancer.

Here, we summarize currently known cell-surface RNA-binding proteins (csRBPs) that have been reported in association with glycoRNAs. NPM1 (nucleophosmin) is a nucleolar phosphoprotein that functions as a multivalent scaffold and chaperone in ribosome biogenesis; it partitions to the granular component of the nucleolus and undergoes liquid–liquid phase separation with rRNA and basic ribosomal proteins (30). Recent work reports that cell-surface NPM1 is present on tumor cells and forms nanoscale clusters near other cell-surface RNA-binding proteins and glycoRNAs, providing imaging-level evidence of organized surface domains. NPM1 represents the only protein with confirmed experimental evidence directly linking it to glycoRNAs at the cell surface (31). A second group, including Nucleolin (NCL), Ro60, and La/SSB, is well-documented as surface-exposed RBPs, but their specific association with glycoRNAs remains to be directly validated (32–34). Finally, several additional proteins, such as hnRNPs, U5 snRNP200 (Brr2), DDX21, HuR, YBX1, ENO1, TLR3/7, have only been suggested by proteomic screens or high-throughput studies, and therefore remain candidates without direct evidence (29, 35). These surface proteins are proposed to regulate intercellular communication via interactions with glycoRNAs, although direct causal roles remain to be established. Beyond the cell surface, glycoRNAs also play central roles in extracellular vesicle (EV)-mediated communication that drives cancer progression (e.g., hnRNPA2B1 and YBX1 proteins) (7). A class of glycoRNAs characterized by terminal sialylated N-glycan structures has been localized to the surface of small extracellular vesicles (sEVs), serving as powerful diagnostic biomarkers. A dual-recognition Förster resonance energy transfer (drFRET) assay was used to screen seven cancer cell lines and identified five prevalent sEV-associated glycoRNAs (U1, U3, U35a, U8, and Y5). In an independent cohort of 100 patients (six cancer types and non-cancer controls), this five-RNA sEV signature distinguished cancer from non-cancer with 100% accuracy (95% CI) and achieved 89% accuracy for cancer-type classification (36). Extracellular vehicles (EVs), including exosomes, microvesicles, and apoptotic bodies, serve as natural carriers for RNA, including glycoRNAs, between cells. For glycoRNAs, the glycan moieties may enhance interactions with lipid microdomains or specific RBPs, facilitating their incorporation into EVs. **Figure 1** summarizes the reported subcellular and extracellular localizations of glycoRNAs, including plasma membrane display and extracellular vesicles, to provide a spatial context for subsequent functional sections. The lipid bilayer protects glycoRNAs from extracellular degradation, while surface glycoproteins and glycocalyx layers on EVs may aid in targeting and recognition by recipient cells, ensuring the effective intercellular transport of glycosylated RNA (7). These observations pertain to glycosylated protein on exosomes rather than to glycoRNAs and are discussed here as a parallel illustrating how glycan-dependent mechanisms on EV surfaces might, in principle, also influence glycoRNA–lectin interactions.

Functional assays further demonstrated that glycoRNAs regulate neutrophil adhesion and transmigration across endothelial cells. When intact neutrophils were treated with extracellular RNase A, which degrades RNAs accessible on the cell surface without permeabilizing viable cells, neutrophil recruitment to sites of inflammation was markedly reduced, consistent with an important contribution of cell-surface RNAs to adhesion and transmigration (37). Neutrophil trafficking into inflamed tissues, including tumors, is known to shape cancer initiation, progression and response to therapy (38). In the present context, we highlight csRNA- and glycoRNA-related regulation of neutrophil recruitment in non-malignant settings for its conceptual relevance to tumor immunology, while noting that comparable mechanisms remain to be directly tested in established cancer models. Across species, the SIDT family encodes conserved double-stranded RNA transport factors: in *C. elegans*, SID-1 is required for systemic RNA interference by enabling cellular uptake of long dsRNA, and in mammals, SIDT2 transports internalized extracellular dsRNA from endolysosomal compartments into the cytosol for innate immune recognition (39–41). These findings not only highlight the biological importance of glycoRNAs but also reveal their critical role in modulating immune cell behavior within the inflammatory microenvironment, underscoring the significance of *Sidt* as a key regulator of this pathway.

## 5 Roles of glycoRNAs in cancer progression

Glycosylation plays a central role in various cellular processes, and aberrant glycosylation is closely linked to multiple disease states. In cancer, dysregulated glycosylation can disrupt signaling pathways, facilitate tumor cell dissemination, and promote immune evasion (42, 43). A recent study has indicated that glycoRNA expression levels may be inversely correlated with tumor aggressiveness in breast cancer models. This stepwise downregulation suggests that loss of glycoRNAs may be associated with the acquisition of more aggressive tumor phenotypes. However, the authors also noted that these observations might reflect correlation rather than causation (16). In the same study, the authors emphasized that these observations are correlative rather than causal and proposed that glycoRNAs may modulate remodeling of the tumor microenvironment by influencing cell adhesion, migration, and interactions with stromal cells; based on the inverse patterns observed in breast cancer models, they further hypothesized that glycoRNAs could restrain invasion and metastasis via analogous pathways (16). These hypotheses remain to be experimentally validated.

In addition to their proposed roles in invasion and metastatic dissemination, glyco-modified RNAs have also been linked to extracellular vesicle–mediated tumor–host communication. Beyond their display at the plasma membrane, glyco-modified RNAs have been detected in extracellular vesicles. Using a dual-recognition FRET assay, Ren et al. identified a small extracellular vesicle (sEV) glycoRNA signature that distinguished cancer from non-cancer plasma and further stratified tumor types (36). More recently, Sharma et al. reported that a subset of small non-coding RNAs within exosomes are themselves glyco-modified; these intraluminal glycoRNAs show enhanced resistance to RNases, can be transferred to naïve recipient cells, and require both exosome biogenesis and intact glycan transfer to proteins for efficient loading into exosomes, indicating a regulated link between protein and RNA glycosylation in EV cargo selection (44). Together with studies on extracellular RNA transporters and glycan-dependent EV cargo sorting (45), these findings support a framework in which plasma-membrane glycoRNAs, vesicle-associated glycoRNAs and non-glycosylated Extracellular RNAs (exRNAs) that rely on glycoprotein machinery represent related but distinct extracellular RNA pools that may cooperate to shape cancer progression and tumor–host communication.

## 6 GlycoRNAs are associated with tumor immune evasion

GlycoRNAs are localized on the cell surface and interact with lectin-like receptors such as Siglecs (sialic acid-binding immunoglobulin-like lectins), transmitting inhibitory signals that help tumor cells evade immune surveillance. Graziano et al. reported that N-glycans on RNA protect endogenous small RNAs from recognition by the innate immune system (3). Removal of N-glycosylation from glycoRNAs derived from cultured cells and circulating sources in both humans and mice triggered strong inflammatory responses, including the production of type I interferons, in a process mediated by Toll-like receptors 3 and 7. Moreover, they found that N-glycans on cell-surface RNAs prevent apoptotic cells from activating endosomal RNA sensors in phagocytes, thereby promoting the non-inflammatory clearance of dying cells. In addition to evidence linking RNA N-glycosylation to immune evasion (16), other studies have provided functional insights into the immunological roles of glycoRNAs. In neutrophils, extracellular RNase A treatment of intact cells, which removes accessible cell-surface RNAs, markedly reduced their recruitment to inflammatory sites *in vivo* and impaired adhesion to endothelial cells *in vitro*, supporting a role for surface glycoRNAs and other csRNAs in efficient immune cell trafficking (37). Furthermore, single-cell spatial imaging of glycoRNAs using the ARPLA method revealed distinct expression patterns across tumor cell lines of varying malignancy, with reduced surface glycoRNAs observed in more aggressive phenotypes. While this study did not directly test immune evasion, the authors documented correlations between glycoRNAs distribution and interactions with monocytes and endothelial cells (16). Tumor-associated sialoglycans on proteins are known to engage Siglecs and transmit inhibitory signals; whether analogous mechanisms apply to glycoRNAs requires further investigation.

## 7 Targeting glycoRNA-related proteins for cancer treatment

A compelling case for targeting glycoRNAs in cancer therapy comes from studies in acute myeloid leukemia (AML). On the cell surface, NPM1 forms nanoclusters with glycoRNAs and other RBPs. These glycoRNA-NPM1 nanoclusters are abundant in AML blasts and leukemic stem cells but are virtually absent in healthy hematopoietic stem cells, making them ideal tumor-specific targets (46). Based on this finding, researchers developed a monoclonal antibody capable of inducing immune-mediated killing of NPM1-positive cells. In preclinical AML models, including patient-derived xenografts, this antibody therapy significantly improved survival and reduced disease burden without damaging normal tissues. Remarkably, this strategy also proved effective against NPM1-positive solid tumors, such as prostate and colorectal cancer models (31). This indicates that targeting the glycoRNA-RBP complex may be a widely applicable therapeutic strategy for both hematological malignancies and solid tumors.

## 8 Combination immunotherapy with glycoRNA manipulation

Effective treatment of multidrug-resistant cancers remains a major clinical challenge. Recent studies revealed that glycosylation contributes to acquired resistance to multiple chemotherapeutic agents. In patient-derived organoids and gemcitabine-resistant cancer cell lines, expression of B4GALT1 was markedly upregulated. This gene mediates N-linked glycosylation of CDK11p110, stabilizing the protein and promoting cancer progression as well as chemoresistance (47). At present there is no direct evidence that B4GALT1-mediated glycosylation of RNA occurs in this context; rather, we highlight this study as an example of how glycan-dependent modification of macromolecules could, in principle, also modulate glycoRNA-associated pathways in cancer. Tunicamycin, a potent inhibitor of glycosylation, has demonstrated significant antitumor activity across multiple cancer types. By inducing endoplasmic reticulum (ER) stress, tunicamycin markedly enhances chemotherapy-induced apoptosis in gastric cancer cells, particularly in multidrug-resistant (MDR) cells. This effect is largely attributable to its inhibition of glycosylation (48). Aberrant glycosylation patterns, such as excessive sialylation, increased fucosylation, and branched N-glycans, have been shown to impair immune recognition and thereby facilitate tumor immune evasion (49). The studies discussed in this subsection primarily investigate glycoprotein rather than glycoRNA pathways. We include them here as conceptual analogies to illustrate how glycan-dependent regulation can influence chemoresistance and immune checkpoint function, while noting that direct evidence for analogous mechanisms acting on glycoRNAs is not yet available. Based on this understanding, the authors propose several potential therapeutic strategies, including enzymatic removal of abnormal glycans (e.g., by sialidases), inhibition of glycosyltransferase activity, blockade of glycan-lectin interactions, and glycoengineering of antibodies or CAR-T cells to enhance immune efficacy. These approaches are considered promising for improving the outcomes of immunotherapy, particularly in combination with existing immune checkpoint inhibitors. However, most of these strategies currently remain at the conceptual or preclinical stage and require further validation. A consolidated summary of currently reported functional roles of glycoRNAs, corresponding readouts, model systems, and primary references is provided in **Table 2**.

**Table 2:** Summary of the functional categories and representative mechanisms of glycoRNAs.

Functional category	Representative mechanism	Biological context	References
Immune Modulation	Binding to Siglec-7/9/11 receptors and regulating innate immune signaling.	NK and macrophage immune regulation.	(3, 37),
Tumor Signaling & RBP Interactions	csRBPs (e.g., NCL, NPM1) form glycoRNA clusters that modulate signaling and adhesion.	Cancer cell surface signaling and immune evasion.	(29, 31),
Intercellular Communication	EV-associated glycoRNAs mediate signaling between neighboring cells.	Extracellular vesicle RNA trafficking and inflammation.	(7, 36),

## 9 Challenges and future directions

Despite growing recognition of glycoRNAs as a promising therapeutic target in inflammation and cancer, research in this area still faces multiple challenges. First, the mechanisms underlying glycoRNA synthesis, trafficking, and functional activity remain poorly defined. Their complex biogenesis involves non-canonical subcellular localization and specific enzymatic pathways that extend beyond classical glycosylation models, and these require further elucidation. Second, therapeutic strategies targeting glycoRNAs currently lack specificity. Existing glycosylation inhibitors act broadly and may interfere with normal cellular functions. Thus, the development of precise delivery systems, such as nanoparticles or aptamer-based approaches, is urgently needed to improve tumor selectivity and minimize off-target effects. Third, the structural complexity and dynamic nature of glycans, coupled with the lack of a simple linear relationship between glycan type and biological function, present significant obstacles. Tumor heterogeneity further complicates the characterization of glycosylation patterns, making the study of glycoRNAs particularly challenging. In terms of clinical translation, current diagnostic methods for glycoproteins often neglect detailed glycan information, limiting diagnostic specificity and utility. Furthermore, targeted therapeutic approaches directed at glycoRNAs as integrated molecular entities have yet to be established.

Recent advances provide promising avenues for addressing these challenges. For example, Zhong and colleagues introduced PRIM-seq (Protein-RNA Interaction Mapping by sequencing), a high-throughput method for unbiased, genome-wide identification of RNA-binding proteins (RBPs) and their associated RNAs (50). Applying PRIM-seq to HEK293T and K562 cells, they constructed a large-scale network, the Human RNA-Protein Association (HuRPA) network, comprising 365,094 RNA-protein interactions involving 11,311 proteins and 7,248 RNAs. Notably, 9,174 of these proteins were previously unannotated, with many predicted by other emerging technologies (e.g., pCLAP, RBDmap) to be potential RBPs. Among the RNAs identified, LINC00339, a long non-coding RNA associated with tumorigenesis, emerged as the largest hub in the HuRPA network. Experimental validation using RNA-proximity ligation assay (RNA-PLA) confirmed all 15 interactions selected for testing, underscoring the robustness of this approach. Future studies should leverage integrative multi-omics strategies combined with spatial transcriptomic techniques (e.g., smFISH, MERFISH) to analyze glycoRNA distribution and functional networks at single-cell and *in situ* resolution. Additionally, research must focus on identifying specific enzymes and key biosynthetic nodes responsible for RNA glycosylation, thereby moving beyond broad-spectrum inhibition to achieve precise targeting. Structure-based drug design and high-throughput screening will be critical for developing therapeutic agents capable of recognizing glycan–RNA complexes. Ultimately, systematic cross-disease comparisons and rigorous preclinical and clinical validation will be necessary to advance glycoRNAs from basic research toward clinical application, paving the way for innovative approaches in cancer precision medicine and drug development.

## 10 Conclusion

As a newly identified class of cell-surface RNA molecules, glycoRNAs challenge the traditional view that glycosylation occurs exclusively on proteins and lipids and open new avenues for exploring RNA-associated glycobiology. Current studies indicate that glycoRNAs can modulate interactions with lectin receptors such as Siglecs and have context-dependent effects on intercellular communication and immune signaling, but their precise roles in the tumor microenvironment are only beginning to be defined. Existing data suggest associations between glycoRNA levels, immune evasion–related pathways and tumor malignancy, as well as potential contributions to extracellular vesicle–mediated communication, yet most of these links are still based on limited models and correlative analyses. Their restricted expression patterns and surface accessibility nevertheless make glycoRNAs attractive candidates for further evaluation as biomarkers for early cancer detection, prognosis assessment and treatment monitoring, and as possible targets for therapeutic intervention. At the same time, major challenges remain, including incomplete understanding of biosynthetic pathways, diversity of glycan structures, tumor heterogeneity and obstacles to clinical translation. Future efforts combining multi-omics, structural analysis and spatial transcriptomics, together with carefully designed functional studies, will be crucial for defining the mechanistic roles of glycoRNAs and for rigorously assessing their potential in precision oncology.
